# Rosai-Dorfman Disease as a differential diagnosis in vocal cord ulceration

**DOI:** 10.1590/S1808-86942010000600021

**Published:** 2015-10-19

**Authors:** Carlos Eduardo Coelho Barbalho, Déborah Nogueira Vasconcelos, João Aragão Ximenes Filho, André Alencar Araripe, Francisco Valdeci de Almeida Ferreira

**Affiliations:** 1 Second-year medical resident in otorhinolaryngology at the Medical School of the Ceará Federal University; 2 Third-year medical resident in otorhinolaryngology at the Medical School of the Ceará Federal University; 3 Otorhinolaryngologist, PhD, adjunct professor of otorhinolaryngology, Medical School of the Ceará Federal University; 4 Otorhinolaryngologist, assistant professor of otorhinolaryngology, Medical School of the Ceará Federal University; 5 Pathologist, PhD, adjunct professor, Ceará Federal University. Walter Cantídio University Hospital - Ceará Federal University

**Keywords:** diagnosis, glottis, histiocytosis, sinus, hoarseness

## INTRODUCTION

Rosai-Dorfman disease (RDD) was described in 1969 by Rosai and Dorfman.^1^ It is a rare benign lymphoproliferative disorder featuring painless neck lymphadenopathy, fever, neutrophilia, an elevated erythrocyte sedimentation rate (ESR), and polyclonal hypergamaglobulinemia.^2^ Published cases describe laryngeal involvement associated with lymphadenopathy or disease in other sites;^3^, ^5^ however, there are no descriptions of glottic involvement only, as in the present case. The etiology of RDD remains unclear. We present a diagnosed and treated case at our unit, and a short review of the literature.

## CASE REPORT

JFG, a female patient aged 58 years, presented with dysphonia during the past four months; there were no other otorhinolaryngological or systemic symptoms. The patient smoked 90 packs or cigarettes per year, consumed alcoholic beverages in social settings, and had no history of tuberculosis or other comorbidities. The physical examination was within normal limits. Videolaryngostroboscopy revealed an ulcer on the right vocal fold and a decreased mucosal wave); laryngeal mobility was normal (Fig. A). Nasofibroscopy was normal. CT of the larynx showed asymmetric vocal folds and no lymphadenopathies. The diagnostic hypotheses were laryngeal tuberculosis or squamous cell carcinoma of the right vocal fold. An excision biopsy was carried out. Histopathology showed non-specific inflammation. Staining for fungi, Koch's bacillus, and leprosy (Grocott and Wade) was unrevealing. A review of the histological slide revealed a mononuclear exudate, histiocytes and giant cells, mostly with emperipolesis. Immunohistochemistry was positive for CD68 and S100 markers in giant cells and histiocytes. Laboratory exams - HIV serology, B and C virus, protein electrophoresis, ANCA, FAN and the rheumatoid factor were within normal limits; the ESR was 22. The PPD, sputum AFB and fungi were negative. Paranasal sinus CT revealed no lesions. A chest CT showed scarring bronchioloectasia in the upper left lobe. Bronchoscopy demonstrated mucosal hyperemia in the upper left lobe, patent ostia and no endobronchial lesions. The tracheobronchial lavage was within normal limits. Laryngoscopy was done every three months for monitoring purposes. No adjuvant therapy was given. The one-year postoperative findings in videolaryngoscopy revealed only minor synechiae in the glottic anterior commissure (Fig. B).

**Figure:**
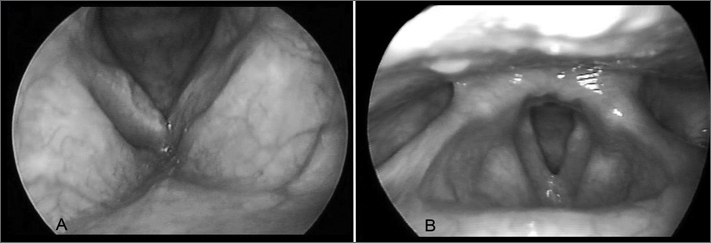
Videolaryngoscopy - (A) An ulcerated lesion on the right focal fold is seen preoperatively. (B) One year postoperatively.

## DISCUSSION

RDD is a rare cause of enlarged lymph nodes; it is found mostly in black males.^3^ The etiology is unclear; an association with immune diseases has been documented.^4^ The clinical picture is not always present.4 The most typical histopathological finding is emperipolesis - lymphocytes or plasma cells surrounded by giant cells. Such giant cells are positive for the S100, a1pha1-antichemotrypsin, and CD68 markers.^2^, ^4^ Extranodal involvement is found in 40% of patients; its diagnosis is challenging, since microscopy reveals more fibroses and less emperipolesis.^5^ In the present case, the patient did not present the typical features of the disease, which complicated the diagnosis. Smoking is a significant risk factor in tuberculosis and laryngeal squamous cell carcinoma; these diseases may present as ulcerative, infiltrative or vegetating lesions in laryngoscopy, and were therefore the initial diagnostic hypotheses. The differential diagnosis of RDD includes lymphoreticular malignancies, AIDS, and idiopathic thrombocytopenic purpura, which have similar histopathological features.^2^, ^4^ RDD is generally self-limiting. Pulsone at al. (2002) found that 50% of cases required no treatment.^6^ These authors concluded that medical observation only is advisable whenever possible, chemotherapy is ineffective, and radiotherapy is of limited use. The role of surgery is to treat obstruction^5^ and carry out biopsies. A study has demonstrated some efficacy of an association between methotrexate and 6-mercaptopurine.^3^

## FINAL COMMENTS

RDD is a rare atypical proliferation of histiocytes. Extranodal involvement alone, as in the present case, is even less common. The etiology is unclear. The diagnosis of RDD is made using histopathological and immunohistochemical methods. The disease progression varies, but is generally self-limited. New therapies are being investigated.
